# Current Evidence on Dietary Patterns and Consumer Food Choices in the Context of Climate and Environmental Change: A Global Perspective—A Report from the Agriculture and Diet: Value Added for Nutrition, Translation, and Adaptation in a Global Ecology (ADVANTAGE) Project Working Group 2

**DOI:** 10.1016/j.advnut.2026.100636

**Published:** 2026-04-15

**Authors:** Lindsey S Taillie, Karen R Siegel, Nicole T Blackstone, Jean M Kerver, Regan L Bailey, Priscilla Connors, Sylvia Rowe, Laina Ewoldt, Constantina Papoutsakis, Daniel J Raiten

**Affiliations:** 1 Carolina Population Center, University of North Carolina, Chapel Hill, NC, United States; 2 Department of Nutrition, Gillings School of Global Public Health, University of North Carolina, Chapel Hill, NC, United States; 3 Hubert Department of Global Health and Emory Global Diabetes Research Center, Rollins School of Public Health, Emory University, Atlanta, GA, United States; 4 Friedman School of Nutrition Science and Policy, Tufts University, Boston, MA, United States; 5 Department of Epidemiology and Biostatistics, College of Human Medicine, Michigan State University, East Lansing, MI, United States; 6 Behavioral Sciences and Social Medicine, College of Medicine, and Institute for Connecting Nutrition and Health, Florida State University, Tallahassee, FL, United States; 7 College of Merchandising, Hospitality, and Tourism, University of North Texas, Denton, TX, United States; 8 SR Strategy, LLC, Washington, DC, United States; 9 Academy of Nutrition and Dietetics, Chicago, IL, United States; 10 Eunice Kennedy Shriver National Institute of Child Health and Human Development, NIH, Bethesda, MD, United States

**Keywords:** climate change, consumer choices, environmental change, food systems, dietary patterns

## Abstract

Climate and environmental change (CEC) are affecting food systems across the globe in myriad ways, yet there has historically been little focus on the role of CEC in influencing dietary patterns and consumer food choices. In this narrative review, we describe the global landscape of dietary patterns and consumer choice to set a baseline understanding of nutrition guidance and estimated food intake. Next, we consider trends in consumer choices across a pathway that includes consumer attitudes, knowledge, and preferences; food purchases and acquisition; food intake behaviors; and food waste. For each step on the pathway, we consider current evidence for how CEC is influencing choices, and identify key questions and data that need to be addressed to inform dietary guidance. We also use a socioecological model to show different levels of policy, community, interpersonal, and individual effects on food intake and their intersection with CEC. We find multifaceted mechanisms through which CEC is likely to influence consumer choices over time. Future dietary guidance should incorporate CEC context to ensure that recommendations are achievable for diverse populations in a food system increasingly likely to be affected by CEC.

**Statement of significance**

The “Agriculture and Diet: Value Added for Nutrition, Translation, and Adaptation in a Global Ecology” (ADVANTAGE) Project is designed to describe our current understanding of how climate and environmental change (CEC) affects diet, nutrition, and health, with a goal toward informing future interventions. This report represents results of the deliberations of the ADVANTAGE Working Group 2 focused specifically on the influence of CEC on dietary patterns and consumer food choices.

## Report Introduction from the Secretariat of the Agriculture and Diet: Value Added for Nutrition, Translation, and Adaptation in a Global Ecology Project

The “Agriculture and Diet: Value Added for Nutrition, Translation, and Adaptation in a Global Ecology” (ADVANTAGE) Project is designed to describe current understanding of how climate and environmental change (CEC) affects diet, nutrition, and health, with a goal toward informing future interventions. The project was organized around the principle that such decisions are based on 3 core sources of evidence: *1*) what is the impact of CEC on priority health targets across the life span; *2*) what is the impact of CEC on dietary patterns and consumer choices; and *3*) what is the nature of the relationships between CEC and food systems [1]. In general, although there is ample evidence to show how dietary patterns and consumer food choices affect CEC via bidirectional interactions with food systems, there is very little evidence to date describing the other direction, i.e., how CEC affects dietary patterns. This report represents results of the deliberations of the ADVANTAGE Working Group (WG) 2 focused specifically on the influence of CEC on dietary patterns and consumer food choices. In this report, research gaps are identified, pointing to specific evidence needed to motivate action in this area.

## Introduction

The global health landscape has become increasingly complex. Diet-related noncommunicable diseases (NCDs)―including obesity, type 2 diabetes, cardiovascular disease, and some diet-related cancers―account for a significant proportion of morbidity and mortality worldwide. In 2017, 1 million deaths around the world were attributable to suboptimal diets, primarily driven by cardiovascular disease [2]. The NCDs described above are largely due to problems of overnutrition (excessive intake of energy). However, many people around the world still suffer from undernutrition (sometimes coexisting with overnutrition in the same household and within individuals). At the same time, susceptibility and response to other global health issues, such as pandemic infections (e.g., vector-borne diseases, HIV, and COVID-19), are exacerbated by poor nutritional status and suboptimal diets.

Each of these nutrition challenges is now compounded by the influence of CEC [3], primarily through complex and diverse effects on the food system. CEC impacts multiple stages of the food supply chain—including production (agriculture, aquaculture, and fishing), processing and manufacturing, distribution and consumption—by altering crop yields, disrupting supply chains, and influencing food availability and affordability. These changes, in turn, affect food purchasing, preparation, storage, and waste at the household level, shaping dietary patterns and consumer behaviors. Consequently, the impact of CEC on food systems directly influences the ability to produce healthy, sustainable foods [4] and ensure good quality diets for the global population.

With growing evidence of the intersection of global health, nutrition, food systems, and CEC, there is a need to support individual-, community-, and policy-level actions toward healthy and sustainable diets―that is, diets/dietary patterns that are good for both human and planetary health. The ADVANTAGE Project aimed to fill that gap, through the work of 4 separate WGs. The objective of this report from WG 2 is to describe evidence on how CEC affects or may affect consumer food behaviors, and thus, dietary patterns, from a global perspective. We aim to understand current dietary choices and the factors that influence them (including CEC-related factors), with the ultimate goals of *1*) informing a targeted research agenda by identifying what we know and what we need to know about these important relationships and *2*) informing organizations responsible for the development and implementation of interventions and policies with available evidence, so such responsible organizations can nudge consumers toward healthier, more sustainable choices within the constraints of our current food system.

We note that although it is evident that consumer choice and dietary patterns also affect CEC (e.g., the production of food affects greenhouse gas emissions, resource depletion, and biodiversity loss) [5,6]because of scope constraints, the focus of this report is on the ways in which CEC affects dietary behaviors. Nutrition guidance should incorporate both of these complex and multidirectional associations (see the ADVANTAGE WG 3 report for more details [4]).

This report is organized in the following way. First, we describe the global landscape of dietary patterns and consumer choice to set a baseline understanding of nutrition guidance and estimated food intake. Next, we consider consumer choice trends and impacts on CEC and identify factors in the food environment or key questions that still need to be answered to adequately inform guidance/policy. We use a socioecological model to show different levels of policy, community, interpersonal, and individual effects on food intake. We also consider consumer food choices along a pathway, e.g., consumer attitudes, knowledge, and preferences; food purchases and acquisition; preparation; intake; and waste.

### Dietary patterns and consumer choice: the global landscape

**FIGURE 1:**
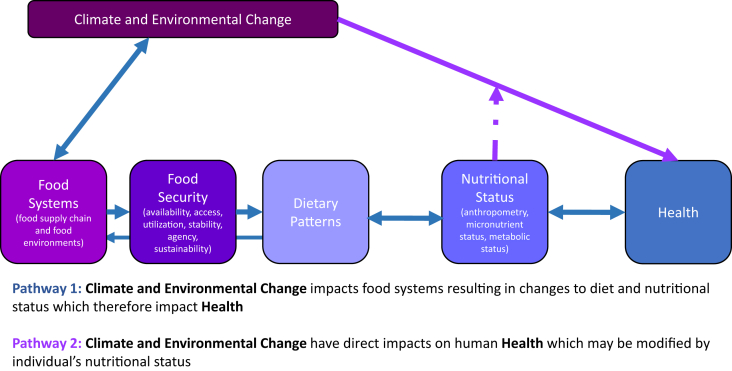
Pathway for climate and environmental change, food (systems and security), dietary patterns, nutritional status, and health. Figure 1 legend: Acknowledgment: This is a modified version of a figure developed by WG1. WG1, working group 1.

A critical locus in the pathway between CEC and human health is human consumption of foods, which is revealed in dietary patterns (Figure 1). These patterns include not only what nutrients and foods people consume, but also how and why they consume them, i.e., in combinations. There are many ways to describe and characterize dietary patterns. For the purposes of this report, we use the global nutrition transition to describe broad global dietary patterns and then focus on 4 key recommended dietary patterns as identified by the United States’ Dietary Guidelines for Americans (DGA) 2020–2025 [7] As an example, we describe overall food intake behaviors of the US population.

The global nutrition transition refers to a complex and significant shift in dietary and lifestyle patterns that has occurred worldwide, particularly in the context of economic development and urbanization [8]. Broadly, the global nutrition transition refers to a shift away from traditional diets toward diets high in animal-source foods like meat and dairy, ultraprocessed foods (UPFs) (e.g., formulations of cheap industrial sources created through a series of processes and usually containing additives) [9], and foods high in added sugar (including sugar-sweetened beverages), fat, salt, and total energy.

Using US dietary intake as an example, the average US diet scores 59 points on the 100-point Healthy Eating Index scale, an aggregate measure of consumption designed to reflect the latest DGA, where a score of 100 indicates perfect alignment with the DGA [10]. Meat consumption in the United States has nearly doubled in the last century, and most meat consumed is red meat (beef, pork, and lamb), whereas nearly a quarter is processed meat [11]. Twelve percent of US adults account for 50% of US beef consumption [12]. American adults have a mean intake of 17 teaspoons of added sugar every day, more than 2–3 times the recommended amount. US diets are also very high in UPFs, with 57% of energy coming from UPFs in 2017–2018, and low in whole unprocessed foods, which account for only 27.4% of consumed energy [13]. In the United States in 2015, 9% of adults were consuming the recommended amount of vegetables, and 12% of adults were consuming the recommended amount of fruit [14].

Similar dietary shifts are observed in low- and middle-income countries (LMICs), with simultaneous large declines in physical activity. In African, Middle Eastern, and Asian countries in particular, a large increase in high-fat foods and edible oils is one of the largest drivers of increased energy consumption, although this is rapidly being replaced by sugar-sweetened beverages and other UPFs [15]. For example, in China, the consumption of edible oil increased from 8.5% to 15.7% of total energy, largely due to an increase in fried foods [16]. Animal-source food intake has doubled from 1991 to 2011 in China and is particularly high in megacities [17]. Food eaten away-from-home and snacking are also becoming increasingly common globally, leading to excess energy intake [18,19].

The changes occurring through the nutrition transition are in stark contrast to nutrition recommendations; that is, diets that are known to help prevent or delay the development of NCDs. For example, the US DGA includes 4 major dietary patterns recommended for health (Table 1), which vary in their inclusion of different food groups, including meat and seafood, but generally include an emphasis on fruits, vegetables, and dairy, and limited intakes of sugar, sodium, and refined starches [7].

**TABLE 1:** Recommended dietary patterns from the US Dietary Guidelines for Americans 2020–2025

Dietary pattern [7]	Description
The Healthy US-Style Dietary Pattern	The Healthy US-Style Dietary Pattern emphasizes fruits, vegetables, whole grains, low- and fat-free dairy, healthy fats, lean meats, and poultry. It limits energy from added sugars and refined starches. The Healthy US-Style Dietary Pattern is based on the types and proportions of foods that Americans typically consume, but it recommends that they be eaten in nutrient-dense forms, prepared with minimal added sugars, refined starches and sodium, and consumed in appropriate amounts.
The Healthy Vegetarian Dietary Pattern	The Healthy Vegetarian Dietary Pattern is a variation of the Healthy US-Style Dietary Pattern that does not include meats, poultry, or seafood. Compared with the Healthy US-Style Dietary Pattern, the Healthy Vegetarian Dietary Pattern is higher in soy products (particularly tofu and other processed soy products); beans, peas, and lentils; nuts and seeds; and whole grains. Inclusion of dairy and eggs would make this dietary pattern an example of a lacto-ovo vegetarian pattern. Like the Healthy US-Style Dietary Pattern, all foods in the Healthy Vegetarian Dietary Pattern are assumed to be in nutrient-dense forms and prepared with minimal added sugars, refined starches or sodium. Foods are also lean or in low-fat forms, with the exception of dairy, which includes whole-fat fluid milk, reduced-fat plain yogurt, and reduced-fat cheese. Within this dietary pattern, there is no recommended amount of energy for additional added sugars or saturated fat, or for more food than what is advised within a particular food group. For this dietary pattern, special considerations are needed for very young children and females who are pregnant or lactating.
The Healthy Mediterranean-Style Dietary Pattern	The Healthy Mediterranean-Style Dietary Pattern highlighted in the DGA is based on the Mediterranean Diet, which focuses on the traditional foods eaten in the countries that surround the Mediterranean Sea. The Mediterranean Diet emphasizes plant-based foods (including fruits, vegetables, whole grains, nuts, and legumes) while incorporating some animal foods—in particular, fish—and includes sweets, red meat, and processed meats only sparingly. The main source of dietary fat is olive oil. This dietary pattern has been associated with many health benefits, primarily related to heart health. When following the Healthy Mediterranean-Style Dietary Pattern, energy up to a specified limit can be used for added sugars, saturated fat, and/or alcohol (for nonpregnant adults of legal drinking age only) or to eat more than what’s recommended for a particular food group.
The DASH Diet	Another dietary pattern received a nod of approval from the 2020–2025 DGA. The DASH Diet, has many of the same characteristics as the Healthy US-Style Dietary Pattern. However, this dietary pattern emphasizes in particular limiting a person’s intake of sodium and saturated fat, and increasing intakes of magnesium, calcium, and fiber (in some cases recommending less sodium than the DGA).

Abbreviations: DASH, Dietary Approaches to Stop Hypertension; DGA, Dietary Guidelines for Americans.

The 2020–2025 Dietary Guidelines were used to create this table instead of the 2025–2030 Dietary Guidelines, which were released simultaneously to the revision of this manuscript and, at the time of writing, subject to controversy in both substance and process by the scientific community. The 2020–2025 Guidelines were also the most recent guidelines to provide a layout detail of dietary patterns.

Despite these recommendations, since the first edition of the DGA in 1980 through today, Americans have fallen short of achieving these dietary goals. For all ages and across both sexes, recommended intakes are suboptimal (except for protein foods, driven by animal-source foods). Overall, 59%–66% of the population exceeds the recommendation for added sugars, 70%–76% exceeds the recommendation for saturated fat intake, and 82%–97% exceeds the recommended sodium intake (females, 95%; males, 97%) [7]. Younger populations (aged 19–30 y) tend to have slightly lower quality dietary patterns as compared with older adults (aged 31–59 y) [7].

There are various potential reasons why so few people are meeting dietary recommendations. Globally, CEC is likely to reduce the potential for regional populations to consume recommended dietary patterns and achieve an overall healthy, sustainable dietary pattern, such as that set out by the landmark 2019 EAT–Lancet Commission on healthy diets from sustainable food systems report [20]. Overall agricultural production (i.e., food availability) may be reduced, and CEC has the potential to hurt global crop yields, thus potentially reducing fruit, vegetable, and grain intake [21,22]. CEC may also increase the occurrence of spoilage and food safety hazards at various stages of the food chain, which may impact food availability as well as human health. These changes decrease the level of macronutrients and micronutrients available in the global food supply, which ultimately impacts the food and nutrients that populations consume [23]. Although animal product consumption is currently high, a wealth of knowledge exists on its deleterious impacts on the environment. Because populations react to this, reduction in animal-sourced protein and a concurrent rise in alternative dietary protein (including plant protein, edible insects, seaweed, microalgae, precision fermented protein, and cell-cultivated) may result, with unclear implications for nutritional status [24]. More broadly, CEC will likely exacerbate nutritional inequities, and marginalized groups may suffer more as their supply chains and food environments are less resilient to shocks [25,26]. More research is needed to understand how CEC is affecting dietary patterns in the United States and globally (see the ADVANTAGE WG 3 report for more details) [4]).

### Consumer food choices in the context of CEC

**FIGURE 2:**
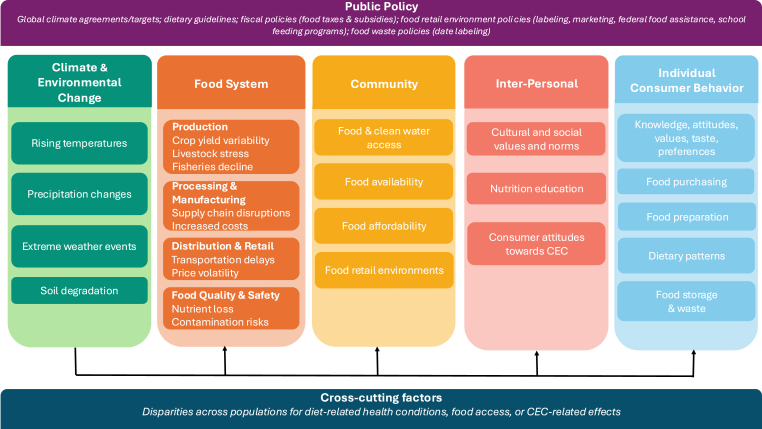
Individual dietary patterns and choices in the context of the socioecological model. CEC, climate and environmental change.

Now that we have described current dietary patterns, it is important to outline some of the drivers of dietary intake and to understand ways in which CEC may affect it. That is, why is population nutrition suboptimal, and how might this change as CEC accelerates? We first describe consumer characteristics (attitudes, knowledge, and beliefs) and cultural characteristics that influence the pathways through which consumers make choices about food, which include: *1*) food purchases/acquisition; *2*) food preparation; *3*) meal practices/food intake (e.g., eating away-from-home compared with home food preparation); and *4*) food waste/storage (Figure 2).

Each subsection considers: *1*) current trends and patterns for this choice; *2*) how the choice (or decision point) is likely to be affected by CEC; and *3*) key questions that need to be answered. Lastly, we outline contextual factors that may influence each step of this consumer decision-making pathway and how these factors may interact with CEC to influence choice.

### Consumer characteristics

#### Knowledge development

Although nutrition knowledge alone is not adequate to improve diet quality, it is positively associated with diet quality and is regarded as an important means of encouraging consumers to make healthy choices [27]. With regard to US consumer knowledge about the environmental impacts of food, a 2019 International Food Information Council (IFIC) survey reported that consumer knowledge remains low, with 60% of survey respondents saying that it is difficult to determine if a food is environmentally sustainable [28]. Despite this uncertainty, >50% of respondents believed it is important for food to be produced in an environmentally sustainable way. In the 2023 IFIC survey, packaging rather than the food product itself was listed as the top way consumers know a product is environmentally friendly. The top packaging characteristics survey respondents reported looking for were “recyclable,” “reusable,” and “made from recycled materials” [29]. Packaging, however, is not generally a leading driver of foods’ environmental impacts, and consumer perceptions of food packaging sustainability may be incorrect [5,30].

In a 2021 survey of food sustainability knowledge among Swiss and German consumers, low knowledge was observed for the timing of local growing seasons and how to balance the environmental impacts of protein sources with transportation methods. The survey further identified that concern about climate change had a small impact on food sustainability knowledge [31]. A systematic review summarizing what is known about consumer food sustainability knowledge in high-income countries concluded that although consumers know about sustainability topics, most consumers do not know how to apply this knowledge to their individual food choices [32]. Taken together, the data suggest that there is a gap in consumer knowledge about the relationship between diet, nutrition, and CEC, and how to translate knowledge into healthy, sustainable food choices. More research will be needed to understand how to effectively communicate this complex topic to diverse populations

#### Consumer attitudes toward climate change

Consumer attitudes toward climate change have been undergoing a significant transformation in recent years, driven by growing awareness of the environmental challenges posed by climate change. Specifically, about a third of US consumers report that climate change is significantly impacting their food choices [29]. There has been a noticeable shift toward greater concern, with many consumers expressing a heightened sense of responsibility for reducing their carbon footprint and mitigating the impacts of climate change. This shift is evident in the demand for more sustainable and ecofriendly products, increased interest in renewable energy sources, and a willingness to make changes in lifestyle choices and adopt more energy-efficient practices [33]. Consumers are increasingly looking to support businesses and brands that align with their climate-conscious values, putting pressure on industries to adopt more sustainable practices and contribute to global efforts to address climate change. This is evidenced by overall sales for products making environmental, social, and governance (ESG) claims having an 8% greater cumulative growth over the 2018–2022 period than their no-ESG claim counterparts [34].

Consumer attitudes toward climate change also vary between countries. A 2022 Yale Program on Climate Change Communication survey found that respondents in Chile, Mexico, Malawi, Bolivia, and Sri Lanka expressed the greatest concern about climate change (65%, 64%, 63%, 62%, and 61%, respectively). The percentage of those in the United States who responded that they are “alarmed” about climate change (34%) trended alongside that of respondents in Canada (35%), Australia (35%), Germany (34%), and the United Kingdom (31%). However, the United States had a much higher percentage of people claiming to be “doubtful” or “dismissive,” with 22% of US respondents indicating these categories compared with 13% in Canada, 16% in Australia, and 11% each in Germany and the United Kingdom [35].

In a model developed by Levi [36] to predict beliefs toward climate change, country-level environmental protection, individual education, and world region were the strongest predictors for awareness and belief in climate change. Variables strongly predicting awareness and belief in climate change did not necessarily have a strong predictive value for the belief in anthropogenic causes of climate change. Rather, regions experiencing more immediate and severe climate impacts were more likely to believe in human causation of climate change [36].

#### Consumer taste preferences

Taste preferences have a profound impact on consumer choices, and are a primary driver of what individuals select and consume [29,37]. These preferences guide decisions at the grocery store, in restaurants, and when cooking at-home. It is not yet clear how CEC will impact taste; however, a handful of studies have reported CEC-induced changes to the taste of foods. Sugiura et al. [38] reported changes to the taste and texture of apples in Japan over the past 30–40 y, likely induced by warmer temperatures. Another study by Ahmed et al. [39] discovered a decrease in perceived tea quality after extreme weather events in China.

#### Culture and social norms

Culture and social norms also shape consumer choices through establishing what communities consider acceptable, desirable, and traditional. There are many ways through which culture and norms about food are transmitted, including familial and household practices, media representations, societal expectations, community traditions, and institutional practices (e.g., in churches and schools). It is likely that as consumer awareness and concern about CEC grow, and as CEC affects water availability and food supply chains, cultural and social norms related to CEC will also affect food attitudes, beliefs, choice, and consumption.

As an example, decades ago in Mexico soda consumption was rare. Over time, the pervasive, aggressive marketing of soda, along with its affordability and widespread availability, created the perception of soda as a modern, desirable product and normalized soda consumption, leading to Mexico having 1 of the highest consumption levels of soda in the world [40]. Although Mexico’s government has attempted to reduce soda consumption through enacting policies like warning labels or taxes, CEC could further normalize soda consumption, as certain regions in Mexico face increased water scarcity and extreme temperatures [41,42]. In addition to exemplifying the complex relationships among the food environment and consumer patterns, the Mexican experience highlights how CEC can affect availability, which will affect choice, and in turn may normalize consumption of certain products over others over time, even after availability issues are resolved. CEC could also affect social and cultural norms in other ways. For example, increased awareness around the environmental impacts of red meat production could change social norms around red meat consumption, resulting in reduced red meat intake in populations with disproportionately high consumption [43,44]. Future research should explore ways in which social norms can be leveraged to promote healthy, sustainable diets. Key questions related to consumer characteristics are listed in Text Box 1.

Text Box 1 **Key questions on consumer characteristics that need to be answered**

**Table:** 

**Key Questions** 1. How are consumer knowledge about and attitudes toward food and climate and environmental change (CEC) changing over time, and how do these influence food choices? Does this vary by country, region, or subpopulation (e.g., adolescents vs. adults)? 2. How are consumer preferences responding to CEC, both in terms of food availability and due to changes in knowledge and attitudes toward CEC? Are tastes for some food groups (e.g., meat and plant-based meat substitutes) changing faster than others? 3. How do changes in food and water availability due to CEC affect social and cultural norms around food and drink, in turn affecting intake? 4. How do social norms around CEC and food proliferate from one group to another group or from one country to another? Is it possible to leverage changing social norms around CEC to increase adoption of healthy, sustainable eating patterns? 5. What are the impacts of CEC on dietary change (e.g., vegan and vegetarian diets, use of plant-based meat/dairy alternatives, low-carbon diets, and use of seasonal produce)? 6. What are the best messaging strategies to communicate to different subpopulations about the intersection of CEC, nutrition, and health?

Alt-text: Text Box 1

### Food purchases and acquisition

#### Current trends in food purchases and acquisition

Acquiring food, typically through purchasing it, is the first step on the pathway of consumers’ choices about what to eat. Food purchases are usually categorized into 2 main areas: at-home and away-from-home. “At-home” foods include purchases of culinary ingredients as well as ready-to-heat and ready-to-eat products that are intended for preparation or consumption at-home. Traditionally, these foods are purchased from food retailers like fresh markets, grocery stores, supermarkets, or big box chain stores [45,46]. In locations with less access to these “traditional” retailers, sources of at-home purchases include convenience stores, corner stores, pharmacies, and dollar stores [47,48]. “Away-from-home” foods include foods that usually do not require additional preparation and are typically consumed outside of the home, including purchases in food service settings (school cafeterias and worksites), institutional settings (hospitals and prisons), restaurants, and fast food.

With regard to current trends in food purchases, in many countries, there have been shifts from people purchasing food predominantly in small fresh markets or in small “traditional” stores (e.g., Tiendas in Mexico) to large big box and supermarket chains [49]. One concern about a potential shift toward increased chain store purchases is that these stores are sometimes associated with purchases of less healthy, more processed foods, both in the United States and other countries [50,51]. With regard to away-from-home food, there has been a global shift in the frequency of away-from-home food consumption [18], which is concerning given that these foods are often higher in energy density and nutrients of concern, such as added sugar, sodium, and saturated fat [52]. US trends in energy consumed from at-home food sources (which includes the full continuum from purchased pre-prepared foods to making complex meals from basic ingredients) compared with away-from-home food sources (e.g., restaurants, fast food, etc.) decreased markedly between 1965 and 2008, with the biggest declines occurring in the 1990s [53].

#### Relationship between CEC and food purchases and acquisition

As previously mentioned, CEC is likely to affect food retail (and hence food purchases) through supply chain disruptions, price volatility, and food quality and safety concerns. CEC may accelerate the aforementioned shift to big box stores if larger retailers are better able to handle the increased variability in the food supply. However, larger retailers with longer supply chains may be disproportionately impacted by CEC.

With respect to food safety, rising temperatures may require food retailers to invest in additional technology to reduce the risk of foodborne illnesses inside stores or restaurants. More broadly, CEC may also make it more expensive for retailers to operate. Some examples of this include extreme temperatures that may increase energy costs for air conditioning or cooling, physical damage to stores due to extreme weather events, or extra resources to comply with new CEC regulations (e.g., California’s new law requiring retailers to donate all surplus foods as part of its strategy to cut greenhouse gas emissions). In addition, CEC could affect food retail by physically reducing access. For example, in Queensland, Australia, a major flood in 2010 not only disrupted the food supply but decreased consumers’ physical access to food retailers [54]. Finally, preliminary evidence suggests an association between rising temperatures and increased purchases of added sugars, primarily attributable to sugar-sweetened beverage purchases [55]. Key questions related to food purchases and acquisition are listed in Text Box 2.

Text Box 2 **Key questions on food purchases and acquisition that need to be answered**

**Table:** 

**Key Questions** 1. In what ways is climate and environmental change (CEC) affecting food retail (e.g., types of retailers; availability/locations of retailers; availability, accessibility, and affordability of foods)? 2. How are potential CEC-related changes in food retail affecting the nutritional quality of food purchases? 3. What strategies will help build resiliency to CEC that will ensure consumers are able to purchase sufficient and healthy foods? 4. In what ways will values, taste, and cost interact to shape consumer behaviors?

Alt-text: Text Box 2

### Food preparation

#### Current trends and patterns related to food preparation

Home food preparation is the behavioral practice that occurs after food is purchased and before food intake. This practice includes cooking, assembling food, and cleaning food preparation areas. More time spent on home food preparation is associated with higher diet quality, less money spent on food outside the home, and less frequent fast-food intake [56,57]. Although older data suggested that home food preparation is decreasing globally, more recent analyses show that the proportion of US adults engaging in home food preparation increased between 2003 and 2016 [53,58]. The greatest increases in US food preparation were seen among males; however, females continue to perform most of the cooking both in the United States and globally [58,59]. US data from 2016 found that 46% of males and 70% of females reported any home cooking, with males and females spending a respective average of 20.4 and 49.5 min/d cooking [58].

#### Relationship between CEC and food preparation

Two recent studies—1 in Sweden [60], [1] in the United States [61]—explored specific barriers to reducing meat intake or increasing intake of environmentally sustainable plant-based proteins. Results indicated that consumer decisions were driven primarily by identity (i.e., personal motivations to cook and the influence of others) and context (i.e., resources including time, money, and equipment). Many participants in both studies expressed a desire to increase intake of plant-based proteins; however, they cited lack of knowledge about recipes or cooking techniques as a major barrier. This example showcases how interventions would need to address multiple domains, such as knowledge, beliefs, and practical skills like food preparation techniques to shift behaviors.

Comprehensive data on appliance usage, including frequency of use of overhead hood ventilation when using gas stoves, is lacking. Recent policy efforts to encourage switching from gas cooking appliances to electric/induction heat sources have faced mixed reception and pushback [62]. Thus, it is critically important to carefully consider communication strategies to facilitate positive action on home food preparation methods as they relate to dietary patterns and CEC [63]. Key questions related to food preparation are listed in Text Box 3 [[63], [64], [65], [66], [67]].

Text Box 3 **Key questions on food preparation that need to be answered**

**Table:** 

**Key Questions** 1. How will climate change awareness affect consumer decisions about food preparation? 2. How will climate and environmental change (CEC)-related factors, such as increasing foodborne disease outbreak frequency [64] and food safety concerns, affect consumer decisions about home food preparation? 3. How can large observational dietary surveys, such as the NHANES [65] or the NIH Environmental Influences on Child Health Outcomes program in the United States [66], as well as other national surveys worldwide, be expanded to include questions that measure home food production and preparation? 4. What are the best ways to teach people cooking methods for improving both health and sustainability, as well as responding to CEC-related changes in the food supply? How can these methods avoid potential unintended consequences of increasing time and effort burden on female household participants [67]?

Alt-text: Text Box 3

### Food intake behaviors

#### Current trends and patterns related to food intake behaviors

Common food intake behaviors are reflected by variation in portion size and differences in eating occasions, such as meals or snacks. The frequency and timing of meals varies across different age, racial, ethnic, cultural, geographic, and socioeconomic subgroups. For example, recent data indicate that Americans average 5.7 eating occasions per day [68] with 85% regularly eating a breakfast comprised <20% of total energy intake per day [69]. In the United States, breakfast consumption is less common among individuals aged 12–19 y, non-Hispanic Black populations, and lower-income populations, whereas Hispanic Americans get more nutrients at breakfast than other racial or ethnic subgroups [70]. Nearly all individuals (93%) report eating dinner, which provides on average one-third (32%–36%) of total energy intake per day. Dinner is less frequently consumed by those aged 12–19 y, Hispanic individuals, and non-Hispanic Black individuals [70].

An examination of trends in adolescent daily breakfast consumption in 23 European countries between 2002 and 2018 showed that more than half of the countries (*n* = 15) had a decrease in daily breakfast consumption, whereas 4 countries had an increase, and 4 had no change. In most of the countries (*n* = 19), daily breakfast consumption was higher among those with higher incomes, and in studies from all of the countries, adolescents with 2-parent households had higher daily breakfast consumption than those in single-parent households [71].

Snacking, or consumption between main meals, is known to influence overall diet quality, as evidenced by a 2018 global review of dietary recommendations in 207 countries/organizations showing that 49 countries and 7 regional or global organizations provided specific recommendations for snacking. Some advised on the quality of snack food, whereas others focused on the frequency of snacking [72]. Snacking is ubiquitous in the United States, with ∼3 in 4 (73%) Americans now snacking at least once per day (compared with 58% in 2021). Most snacking is reported to occur between lunch and dinner or after dinner, providing about one-quarter (23%) of total energy intake per day [73]. In China, the China Health and Nutrition Survey showed an increasing trend of energy derived from snacks between 1991 and 2018 with females, younger individuals, and urban residents having a higher proportion of energy from snacks compared with meals [74].

Attention is often directed at an increase in portion size as a contributor to the obesity epidemic. Conversely, increased awareness of portion sizes has been recommended to promote increased consumption of fruit, vegetables, and pulses, and decreased consumption of high-energy-dense foods [75]. Because portion sizes can vary substantially by country or cultural group, scientists in a group of European countries have suggested harmonizing standard reference portions, especially for vegetables, legumes, fish, and dairy. A trend analysis of portion sizes of commonly consumed discretionary foods among Australians from 1995 to 2011–2012 showed an increase in portion size for 7 out of 14 food categories (pizza, cake, sausage, cereal bars, processed meats, ice cream, and wine). Portion size decreased for 3 food categories (pastry, snack food, and fried potatoes), and portion size remained the same for 4 categories (biscuits, chocolate, sugar-sweetened beverages, and beer) [76].

#### Relationship between CEC and food intake behavior

Ambient temperature may play an important but little-known role in food intake behavior. Results from a Chinese study of associations between ambient temperature and food intake using ∼1 million food purchasing receipts showed that an increase in temperature of just 1°C was associated with a 0.11% decrease in food intake, with a greater change among females than males. Further analyses showed a U-shaped relationship between ambient temperature and ordering takeout food, with both higher and lower temperatures associated with an increase [77]. In another study that assessed the impact of shifts in annual precipitation and temperature on UPF intake in Mexico, 0.5 mm annual decrease in precipitation and 0.1°C annual increase in temperature were associated with a 0.011% and 0.03% increase in mean daily energy from UPFs, respectively [78]. Key questions related to food intake are listed in Text Box 4.

Text Box 4 **Key questions on food intake that need to be answered**

**Table:** 

**Key Questions** 1. How might climate and environmental change (CEC) impact people’s priorities when deciding which foods to consume and when? 2. How might people’s priorities change across the life course or across different demographic subgroups? 3. How and why might CEC impact taste preferences for certain foods? 4. How will CEC impact the consumption of animal-source foods compared with alternative dietary protein sources? 5. How might different CEC impacts (e.g., time course of weather, disasters, other environmental factors) affect preferences?

Alt-text: Text Box 4

### Food waste and storage

#### Current trends and patterns in food waste and storage

In 2019, the FAO of the United Nations reported that, globally, 14% of food was lost between harvest and market, and 17% was wasted at the retail level and by consumers [79]. Consumer food waste is defined as edible food appropriate for human consumption; that is, discarded or left to spoil for any reason [80]. Per-capita, high- and middle-income countries have greater consumer waste rates than low-income countries. For example, consumers in Europe and North America waste 95–115 kg per-capita annually, whereas consumers in sub-Saharan Africa and Southeast Asia waste 6–11 kg [81].

Despite significant data limitations [82], recent studies have examined global food waste patterns to estimate the relationship between per-capita income and food waste [[83], [84], [85]]. For example, Lopez Barrera and Hertel [84] found that the share of wasted food is low in low-income countries but rises rapidly as per-capita income increases before converging to a relatively stable level of 29%–36%. Verma et al. [85] similarly found a baseline threshold of consumer expenditure ($6.70 per-capita per day) beyond which food waste rises rapidly and then levels off at high levels of affluence. In the United States specifically, recent estimates indicate that consumers waste 0.42 kg of edible food each day [86], with total consumer loss valued at $371 per-capita per year or 9.2% of the average consumer food expenditure [87].

Consumer food waste behaviors are influenced by numerous, often context-specific factors, though some common drivers have been identified. Improper storage, confusion regarding the meaning of date labels [88], and ineffective purchasing and storage practices are common causes of waste at the consumer level [89]. Changes in food appearance, palatability, and perceived safety impact consumer acceptance and utilization. In a study of US households, Dusoruth and Peterson [90] found an increased likelihood of consumers discarding foods when appearance deteriorated, suggesting that skills in differentiating between edibility and cosmetics have the potential to reduce waste.

#### Relationship between CEC and food waste and storage

Global CEC portend weather extremes with detrimental effects on food storage for both hot and cold weather. Risks of hot weather events include increased microbial growth, undesirable appearance and palatability changes, reduction in nutritional value, increased rancidity of fats and oils, and a shortened shelf life for both processed and unprocessed foods [91].

Maintaining a recommended cold storage temperature for perishable foods is critical for both safety and quality. In a study of home food storage practices, Ovca and Jevšnik [92] found that most consumers failed to monitor refrigerator temperatures and shelved foods, having no regard for cross-contamination potential. Convenience took precedence over food safety and optimizing the cooling capacity of the refrigerator.

Although cold weather seems more favorable to food storage, foods high in water content undergo changes in texture because of crystallization, and in crispness because of structure changes and cell damage [93]. Prolonged cold deteriorates flavor and increases the risk of undesirable odor absorption in stored foods. Repeated freeze and thaw events alter the mineral content of food because of the expansion and contraction of ice crystals [94]. These events also increase incidences of food packaging failure because of expansion and contraction of contents, damage to seal integrity, brittleness of package materials, and moisture migration. Key questions related to food waste and storage are listed in Text Box 5.

Text Box 5 **Key questions on food waste and storage that need to be answered**

**Table:** 

**Key Questions** 1. Do knowledge or values around climate and environmental change (CEC) influence consumers’ food waste patterns? This could include wasting less or managing waste differently (e.g., composting). 2. Is perishability or “food waste potential” a barrier for increasing consumption of healthy foods, like fresh fruits and vegetables, for low-income households? If so, could this be exacerbated by CEC for subpopulations who lack access to refrigeration or air conditioning? 3. What will be the role of technology in preventing food spoilage, reducing food waste, and promoting food safety in households in the future?

Alt-text: Text Box 5

### Contextual factors influencing the process of food acquisition, preparation, intake, and waste/storage

#### Food availability

Food access and availability are well-established drivers of food choice [95]. In short, consumers must have access to available foods and drinks to select and consume them. CEC will affect foods available for consumers by impacting the quantity, quality, and timing of food as it moves through the food supply [96]. Gradual shifts in average conditions as well as acute disturbances (e.g., increasing frequency and severity of extreme weather) will affect food across all stages of the food supply chain (agricultural productivity, storage, processing, distribution, and retail) [96]. Some regions are likely to be more affected than others, with predictions that CEC’s impact on agricultural productivity will likely be highest in tropical regions, including many of the world’s poorest countries [97,98]. In communities with lower access to global markets and where food consumption is more tightly linked to food production (i.e., short supply chains), CEC could lead to shortages of specific foods [96]. Potential implications include reduced dietary diversity, increased food insecurity, and potentially increased vulnerability to adverse health outcomes associated with the nutrition transition.

Other evidence suggests that it is not just the availability of food but also the quality of food that will be affected (e.g., diversity, nutrient density, and safety) [99]. For example, the increase in atmospheric carbon dioxide could have a fertilization effect on some crops (e.g., soybean, wheat, and rice), while simultaneously reducing mineral concentrations [4]. The overarching effects of concurrent CEC changes on nutrient quality in the food supply are still an area of emerging research [100]. Other changes to quality relate to freshness and food safety, with concerns that higher temperatures will lead to more rapid decomposition of produce and facilitate the growth of pathogens.

Lastly, CEC-linked food shortages combined with the concomitant spread of transnational corporations may accelerate the nutrition transition to UPFs. For example, a recent study in Mexico found that decreases in rainfall and increases in temperature were associated with increased intake of UPFs in tropical regions [78].

#### Water availability

In addition to concerns about food availability, CEC is likely to adversely affect water availability, including both the quantity and quality of water available for individual consumption and use. Modalities through which CEC is expected to do this include increased water contamination, groundwater depletion, and freshwater salinization [101]. Concurrently, hydration needs may increase in areas experiencing increased heat, increased diarrheal disease, or other vector-borne diseases that may increase as a function of CEC [102]. Reduced water availability has many potential effects on dietary patterns, as adequate clean water is needed not only for drinking but also for food preparation, sanitation, and the feeding of infants and young children [103]. Moreover, a lack of acceptable household tap water could reduce diet quality if individuals eat more meals away-from-home, select substitute sugar-sweetened beverages over alternative water options, or cannot clean fresh produce [[103], [104], [105]]. The negative impacts of water insecurity are experienced in both high-income countries and LMICs; however, as with other CEC-fueled crises, the burden is greater for those of lower socioeconomic status [106,107]. Ensuring water security for all will require structural changes; however, methods for adapting to water scarcity such as water desalination, wastewater treatment, and new well drilling often require significant upfront investment that makes them inaccessible to many low-income communities [[108], [109], [110]].

#### Food price/cost

CEC is likely to affect food prices. Along with availability, food price is a major driver of food choices across the globe [111,112]. People with a low socioeconomic status tend to be more price sensitive [113]. Across the globe, poor quality foods and diets tend to be cheaper on a per-kcal basis and are important drivers of socioeconomic status disparities in dietary quality [114,115]. More recently, additional data suggest that, compared with unprocessed foods, UPFs have lower nutrient density, higher energy density, and lower per-kcal cost [116]. In addition, they have faced slower price increases in recent years. Although this relationship is better established in high-income countries, in LMICs, the relationship between cost and healthfulness of diets is more mixed [[117], [118], [119]] and the relative cost of fruits and vegetables tends to be less expensive than in high-income countries [120]. This relationship is made more complex by the fact that several countries, such as Mexico, Colombia, and Hungary, have already implemented taxes on unhealthy products like sugar-sweetened beverages and UPFs [121], which by design disproportionately raises prices on these items.

These underlying trends regarding price and healthfulness of diets are concerning when juxtaposed with recent increases in global food prices because of extemporaneous events such as the COVID-19 pandemic and international conflict [122]. CEC is likely to accelerate trends toward increasing food prices as climate change creates more favorable conditions for disease spread or other shocks to supply (e.g., heat stress reduces poultry production; see ADVANTAGE WG 3 report for more examples [4]).

A recent meta-analysis predicts global food price increases of around 30% until 2050, with disproportionate price increases in Africa and Asia compared with North America [123]. Major questions to address with future research relate to what types of foods will be most affected by CEC-related price increases, which regions and subpopulations will be most affected, and how these price changes will influence the nutritional quality of diets.

Although CEC will affect underlying food prices, it is less clear how consumers will make tradeoffs between CEC, price, and other factors. Price is highly likely to be a limiting factor as consumers consider a shift to more CEC-friendly options. Indeed, a recent study found that prices were a barrier to making healthy and sustainable food choices for parents in Belgium, particularly parents with low socioeconomic status [124].

#### Marketing and advertising

It is well established that food marketing is a major driver of food choices, by affecting consumer attitudes and preferences not only for the marketed products but entire categories of products. Although CEC is not expected to affect the ability of companies to market foods, companies may leverage CEC concerns to market foods to increase consumer preferences and purchases. Of particular concern is “greenwashing,” or the use of potentially misleading or unsubstantiated claims or marketing techniques that make a product seem more sustainable than it is [125]. Examples include the use of nature-based imagery or colors, environmental claims or certifications that have not been created or implemented by an independent third party, and corporate social responsibility campaigns [125]. There is likely heterogeneity in the use of these techniques by country, region, and demographic, as some consumer segments are more responsive to concerns about CEC than others (e.g., younger consumers) [29]. Another key question is whether the presence of environmental marketing techniques will lead to a “halo effect,” where consumers overgeneralize the positive attributes of a product (e.g., they might think a product is more nutritious than it actually is) [126]. Research in this space is needed to understand who is most affected by CEC-related marketing and the role it plays in consumer preferences, choices, and the healthfulness of purchases.

In addition to product promotion, CEC is already playing a role in influencing the creation of new products. Companies are developing new technologies and products to commercialize foods with higher environmental sustainability. The most well-known of these are alternative nonanimal-source proteins, including nondairy milks and biomimicry meat products. There has been rapid growth in sales for both types of products over recent years, although the market for meat alternatives in the United States appears to remain small [127]. One major concern is the extent to which substituting with these products affects nutrient intake. For example, nondairy milks are not recommended for consumption by young children without medical indications, because they are not nutritionally equivalent to cow’s milk and often contain added sugar [128]. With regard to meat substitutes, some scholars have pointed out that many of these substitutes are high in sodium and ultraprocessed [24]. Others have raised concerns around ethical issues, such as consuming products grown from cell cultures [129]. Additional research is needed to understand trends in purchases and intake of these products, their association with diet quality, and who is consuming them and why.

#### Food labeling

Food labeling influences consumer choices by providing information about nutritional properties and using visual communication strategies to influence consumer attitudes and choices [130]. As consumer awareness of CEC has grown, there has been a proliferation of CEC-related food labels on food packages, ranging from numeric labels (i.e., carbon emissions associated with a product) to interpretive labels (e.g., an ecoscore indicating the relative ecofriendliness of a product) to claims and certifications [131,132]. Because these labels are relatively new, there is a paucity of data about how consumers understand or use them, with existing data suggesting heterogeneity by geography and socioeconomic status [[131], [132], [133]]. However, rapid growth in the number and type of labels, coupled with the lack of standardization, suggests that consumers may be confused about how to interpret and use these labels. Some scholars and advocates posit that, much as consumers have a right to know what is in their food with respect to nutrients and ingredients, consumers have a right to know about the environmental impacts of their food [31]. One option to address this is to require a more transparent and rigorous process for standardizing environmental labels on food packages. Another option would be to require standardized front of package labels disclosing when products have high levels of environmental harm, such as greenhouse gas emissions. Some consumer research has shown that, similar to nutrition and health labels [134], such labels are well-understood and influence consumers’ selection of products [135,136]. However, the legal and regulatory feasibility of these types of labels likely varies by country. Additional data are needed to understand the psychological mechanisms of CEC-related labels, how they influence food choice, and who is most likely to use and understand them. Food labels are also relevant to consider with regard to food waste, as labeling systems on expiration dates in some countries are confusing to consumers, potentially leading to greater levels of food waste [137,138]. Key questions related to these contextual factors are listed in Text Box 6. A summary of all identified priorities for future research is listed in Table 2.

Text Box 6 **Key questions on contextual factors that need to be answered**

**Table:** 

**Key Questions** 1. What policy approaches work to improve food and water availability in the context of climate and environmental change (CEC)? 2. Can new or updated packaging labels better inform consumers about the environmental impact and nutritional quality of products to alter their food choices? 3. What policy approaches work to help inform consumers about the amount of climate and/or environmental harm associated with food production and consumption?

Alt-text: Text Box 6

**TABLE 2:** Priority areas for future research

Area	Priority areas for research
Consumer characteristics	• Documenting shifts in consumer knowledge, attitudes, preferences, and norms about CEC and food choice, overall and by region and subpopulation • Evaluating the association of CEC with food choices and dietary behaviors • Developing effective messaging strategies about CEC, nutrition, and health
Food purchases and acquisition	• Assessing and building resiliency to impacts of CEC on food access, availability, and affordability • Evaluating the association of CEC with the nutritional quality of food purchases • Identifying how CEC-induced shifts in consumer dietary preferences and values alter purchasing behaviors
Food preparation	• Evaluating how factors such as CEC awareness or food safety concerns alter food preparation behaviors • Pursuing opportunities to incorporate assessment of food preparation into large observational surveys • Investigating associations between CEC and various food preparation behaviors, and educating consumers on ways to prepare food in methods resilient to CEC-related changes
Food intake	• Evaluating the association of CEC with food and water insecurity and dietary patterns • Assessing how shifting priorities across life groups and demographics impacts the foods selected for intake, including protein sources • Quantifying impacts of CEC events, such as extreme weather events, on dietary intake and elucidating pathways
Food waste and storage	• Identifying drivers of food waste patterns, such as knowledge, values, or access to preservation methods • Determining how concerns about increased foodborne illness or waste influence food decisions and thereby diet quality • Developing and improving technologies aimed at extending shelf life of foods and limiting food waste

Abbreviation: CEC, climate and environmental change.

In conclusion, the relationship between consumer food behavior and CEC is bidirectional, meaning that they mutually influence each other in a complex and interconnected manner. On one hand, consumer food behavior significantly impacts CEC. On the other hand, CEC has a profound influence on consumer food behavior, which is the focus of this paper. As the climate shifts, it can disrupt food production and supply chains, leading to changes in food availability, quality, and price. Extreme weather events, such as droughts and floods, can affect crop yields and food security, potentially influencing which foods are accessible and affordable to consumers. Growing awareness of climate change and its impacts can lead to shifts in consumer attitudes and preferences. People may become more conscious of the environmental consequences of their food choices and seek more sustainable and climate-friendly options. A coordinated effort by researchers, policymakers, businesses, and individuals is needed to promote sustainable and climate-resilient food systems that not only reduce the environmental impact of food consumption but also adapt to the challenges posed by a changing climate.

## Author contributions

The authors’ responsibilities were as follows – DJR: conceptualized the study; LST, KRS, CP: further refined the study concept with input from all coauthors and drafted the manuscript; and all authors: contributed to the writing of subsequent manuscript drafts and read and approved the final manuscript.

## Funding

LST’s time was supported by funding from Bloomberg Philanthropies. RLB is funded through NIH/NCI R01CA215834.

## Declaration of Generative AI and AI-assisted technologies in the writing process

The author(s) declare that no generative AI or AI-assisted technologies were used in the writing of this manuscript.

## Conflict of interest

The authors report no conflicts of interest. The NIH has had previous contracts with the Academy of Nutrition and Dietetics. The contents of this article represent the authors’ views and do not constitute an official position of the NIH or the United States Government.
